# Percutaneous Implantation of a Microcatheter-Port System for Hepatic Arterial Infusion Chemotherapy of Unresectable Liver Tumors: Technical Feasibility, Functionality, and Complications

**DOI:** 10.3390/diagnostics11030399

**Published:** 2021-02-26

**Authors:** Olivier Chevallier, Ségolène Mvouama, Julie Pellegrinelli, Kévin Guillen, Sylvain Manfredi, François Ghiringhelli, Nicolas Falvo, Marco Midulla, Romaric Loffroy

**Affiliations:** 1 Department of Vascular and Interventional Radiology, Image-Guided Therapy Center, François-Mitterrand University Hospital, 14 Rue Paul Gaffarel, BP 77908, 21079 Dijon, France; olivier.chevallier@chu-dijon.fr (O.C.); segolene.mvouama@chu-dijon.fr (S.M.); julie.pellegrinelli@chu-dijon.fr (J.P.); kguillen@hotmail.fr (K.G.); nicolas.falvo@chu-dijon.fr (N.F.); marco.midulla@chu-dijon.fr (M.M.); 2 Department of Gastroenterology and Hepatology, François-Mitterrand University Hospital, 14 Rue Paul Gaffarel, BP 77908, 21079 Dijon, France; sylvain.manfredi@chu-dijon.fr; 3 Department of Medical Oncology, Georges François Leclerc Center, 1 Rue Professeur Marion, 21000 Dijon, France; fghiringhelli@cgfl.fr

**Keywords:** liver cancer, hepatic arterial infusion, intraarterial chemotherapy, port catheter, percutaneous implantation

## Abstract

To evaluate the feasibility and safety of percutaneously implanted arterial port catheter systems for hepatic arterial infusion of chemotherapy (HAI) in patients with unresectable liver malignancies. From October 2010 to August 2018, arterial port catheters for HAI were percutaneously implanted in 43 patients with unresectable liver malignancies. Three different catheter placement techniques were compared: a conventional end-hole catheter placed in the common hepatic artery (technique 1, *n* = 16), a side-hole catheter with the tip fixed in the gastroduodenal artery (technique 2, *n* = 18), and a long-tapered side-hole catheter with the tip inserted distally in a segmental hepatic artery (technique 3, *n* = 6). Catheter implantation was successful in 40 (93%) of the 43 patients. Complications related to catheter placement were observed in 10 (23%) patients; 5 (83%) of the 6 major complications were resolved, as well as all 4 minor complications. Catheter migration and occlusion occurred in 9 (22.5%) patients. Catheter migration was more frequent with technique 1 (*n* = 6) than with technique 2 (*n* = 1), although the difference was not significant (*p* = 0.066). Percutaneous arterial port catheter implantation for HAI is highly feasible and carries a low risk of complications.

## 1. Introduction

The liver derives its blood supply from two sources; namely, the arterial circulation and the portal circulation [1,2]. Due to the portal venous drainage, it is a predominant site for metastases from colorectal cancer. However, the main source of blood supply is the hepatic artery for liver metastases and primary liver cancers, whereas the portal circulation chiefly supplies the normal liver cells. Regional hepatic arterial infusion of chemotherapeutic agents (HAI) allows the direct delivery of drugs to the liver tumors via the arterial supply, achieving high concentrations of antineoplastic agents in the tumors while limiting the toxicity to untargeted organs [3,4]. HAI has demonstrated its effectiveness not only for unresectable colorectal liver metastases but also for primary liver cancer and for metastases from other primary malignancies [4,5]. 

The implantation of permanent intra-arterial port catheter systems was initially performed surgically. This method was invasive and exposed patients to the risks associated with laparotomy and general anesthesia. Since the 1980′s, minimally invasive percutaneous techniques have allowed easier and safer port catheter implantation, with no laparotomy or general anesthesia [4]. However, percutaneous port catheter placement may be challenging, especially in the presence of variant hepatic arterial anatomy [6]. The operator must be highly experienced to minimize the risk of procedural failure. In addition, secondary catheter migration can occur, leading to extrahepatic drug infusion, which carries a high risk of complications [7]. The various catheter placement techniques described to date are associated with different catheter migration risk levels [1,5,8]. The aims of this study were to determine the feasibility and safety of percutaneous port-catheter system insertion for HAI in patients with unresectable liver malignancies.

## 2. Materials and Methods

### 2.1. Study Population 

We retrospectively reviewed all percutaneous catheter-port system insertion procedures for HAI performed in our hospital from October 2010 to August 2018. In all patients, the decision to use HAI was taken during a multidisciplinary meeting. Each patient received detailed information about catheter placement and HAI chemotherapy. Informed consent to the procedure was obtained from each patient before the procedure. This study was approved by our institution’s ethics committee, which waived the need for informed patient consent in compliance with French legislation on retrospective studies. Inclusion criteria were one or more liver tumors not amenable to surgical resection, performance status of 2 or less in the Eastern Cooperative Oncologic Group classification (ECOG), and a normal bilirubin level. Exclusion criteria were significant extrahepatic disease, life expectancy of less than 3 months, and presence of ascites or liver failure. 

### 2.2. Percutaneous Microcatheter-Port System Implantation

A tapered Anthron^®^ P-U non-stretched 5 French catheter (Toray, Chuo City, Tokyo, Japan) was connected to a subcutaneously implanted Celsite^®^ port (B. Braun Medical, Melsungen, Germany). All procedures were performed in angiography suites under local anesthesia with lidocaine, by 4 different interventional radiologists with more than 5 years of experience in interventional and vascular radiology. Port catheter placement was performed in one or two stages. (1) For the one-stage procedure, diagnostic mesenteric and celiac angiograms were obtained for hepatic arterial road mapping [8]. Then, if necessary, arterial redistribution was performed to convert multiple hepatic arteries into a single arterial supply. In addition, extrahepatic arterial branches arising from the hepatic artery and supplying extrahepatic organs were occluded to prevent nontarget drug delivery [8]. Finally, the catheter-port system was implanted. (2) For the two-stage procedure, the catheter port-system was implanted in a second session one or two weeks after arterial redistribution and/or extrahepatic arterial branch embolization. Occlusion of new or initially missed extrahepatic arterial branches discovered during this second session was performed when necessary.

The procedure was performed as follows: a 5-Fr catheter was inserted in the common femoral artery using the Seldinger technique [5]. Angiographies of superior mesenteric and celiac trunk were obtained for arterial road mapping. A microcatheter was then inserted coaxially for artery embolization. When an aberrant hepatic artery was identified, hepatic arterial blood flow was redistributed using microcoils and/or N-butyl cyanoacrylate (NBCA; Glubran^®^2, GEM Srl, Viareggio, Italy) mixed with iodized oil (Lipiodol, Guerbet, Villepinte, France) in a 1:1 ratio to convert multiple hepatic arteries into a single arterial blood supply. If necessary, extrahepatic arterial branches arising from the hepatic artery and parasitic arteries with hepatopetal blood flow were also occluded. In the absence of hepatic artery anomalies, the gastroduodenal arteries and almost all right gastric arteries were embolized with microcoils to avoid extrahepatic diffusion of the chemotherapeutic drug. The port catheter system was implanted during the same procedure (one-stage procedure) or one week later (two-stage procedure) using the catheter-exchange method on a 0.014″ supportive delivery guide wire. If the femoral approach was not achievable, the catheter was inserted into a branch of the subclavian artery [9]. 

**Figure 1:**
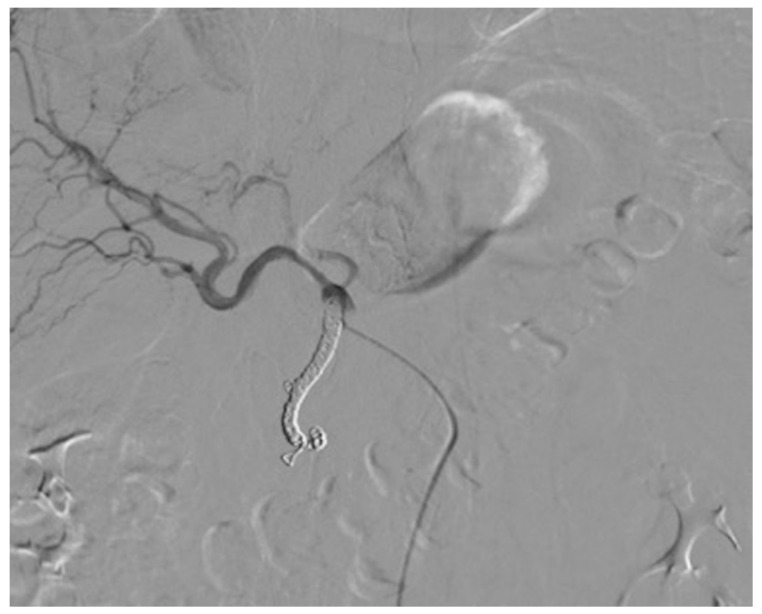
Technique 1. The tip of the catheter with its end hole is simply inserted into the common hepatic artery.

**Figure 2:**
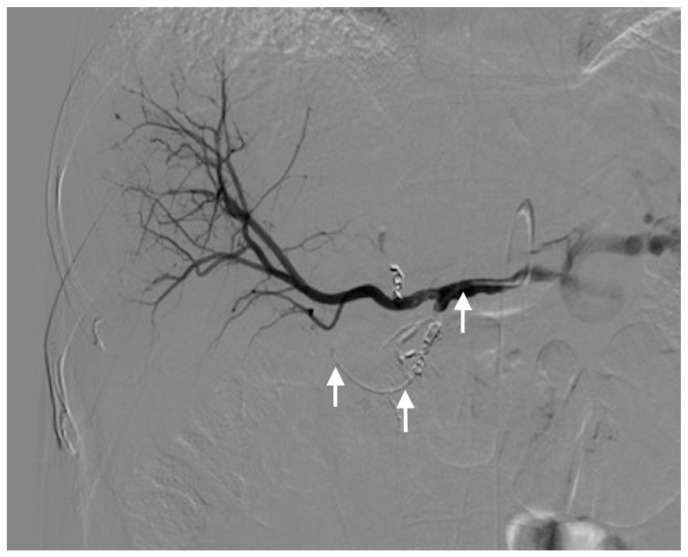
Technique 2. Final hepatic angiogram after embolization of the gastroduodenal artery and right gastric artery. The catheter is well visualized (arrows).

**Figure 3:**
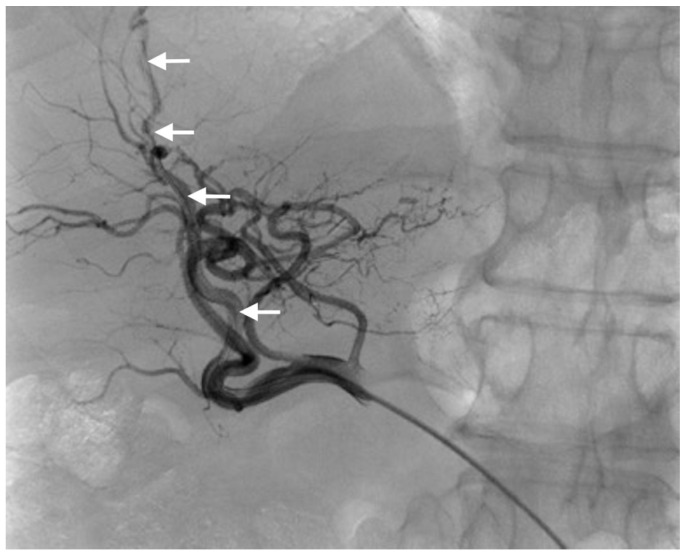
Technique 3. Final angiogram after catheter placement in a segmental branch of the hepatic artery (arrows).

Three different techniques were used for catheter placement [1,10]. (1) Technique 1, free tip catheter placement: the gastroduodenal artery and right gastric artery were first embolized using microcoils. Then, the catheter was cut above its side-hole and the tip was simply inserted into the common hepatic artery. The chemotherapeutic drug was then infused into the artery through the end-hole of the catheter (Figure 1); (2) Technique 2, original fixed-tip method: a side-hole catheter was implanted into the hepatic artery. Its side hole was then placed in the distal part of the common hepatic artery before the origin of the gastroduodenal artery. The distal catheter shaft was inserted and fixed within the gastroduodenal artery by microcoils. The microcoils were delivered through a microcatheter inserted into the indwelling catheter and reached the gastroduodenal artery through its side-hole (Figure 2); (3) Technique 3, used as a back-up plan: a long tapered side-hole catheter was introduced into the hepatic artery. The catheter tip was then placed distally into the segmental hepatic artery, with its side hole placed in the proper hepatic artery (Figure 3).

**Figure 4:**
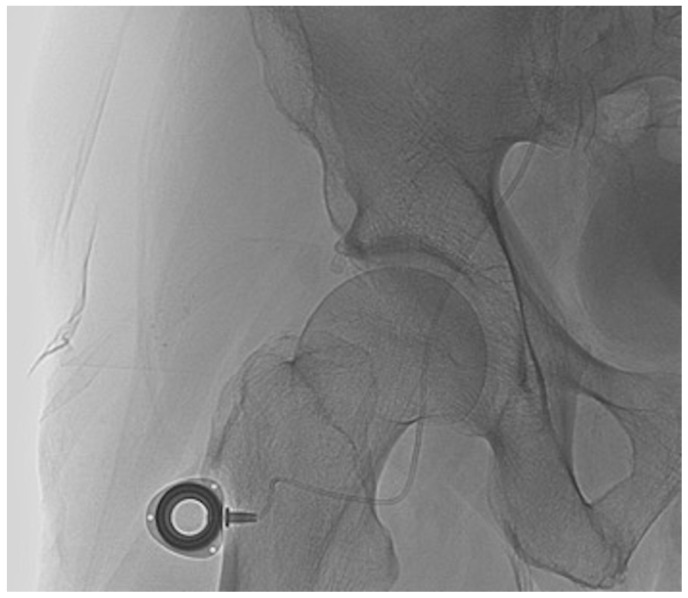
The port chamber in the groin area whatever the technique.

Finally, an approximately 1-cm long transverse skin incision was performed distally to the groin at the anterior surface of the thigh to implant the port (Figure 4) [10]. A dilator was then inserted caudally to make a subcutaneous tunnel at a depth of about 0.5 cm in the subcutaneous fat layer [10]. The external part of the port catheter was shortened from the incision site and connected to the port chamber. The incision site was closed with simple interrupted suture. Port catheter angiography was performed using a 19-gauge Huber needle to confirm the proper positioning of the catheter side hole or tip. The port was then flushed with 1 mL of heparin mixed with 9 mL of saline to prevent catheter thrombosis. If the femoral approach was unsuccessful, the catheter was inserted into a branch of the subclavian artery. After the procedure, patients were asked to remain in bed for 6 h and were kept under medical supervision for at least 24–48 h. No prophylactic antibiotics were administered.

### 2.3. Functional Assessment of the Port Catheter

**Figure 5:**
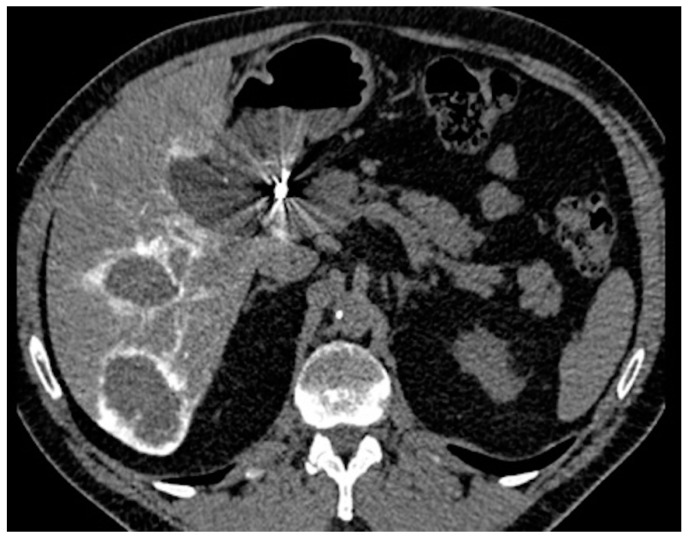
CT scan with contrast injected through the arterial port catheter.

Non-contrast-enhanced and arterial phase-enhanced abdominal CT scans were obtained for all patients about 1 week after catheter implantation, with 10 mL of contrast medium followed by 20 mL of saline injected at 0.5 mL/s through the arterial port catheter, to detect catheter migration or extrahepatic perfusion (Figure 5). The port catheter was flushed with 1 mL of heparin mixed with 9 mL of saline to avoid catheter thrombosis. No reflux test was performed because of the risk of catheter thrombosis [10]. Strict aseptic conditions were maintained. Contraindications to enhanced CT scan were cutaneous infection, collected hematoma, contrast medium allergy, and severe kidney failure. 

### 2.4. Intraarterial Chemotherapy Treatment

HAI was started a few days after the procedure, depending on the clinical circumstances and on the results of the CT scan. Patients received their chemotherapy only if no extrahepatic perfusion or catheter migration was seen on the 1-week control CT-scan. When a complication occurred, such as hematoma, infection, catheter migration, or dysfunction, the first course of chemotherapy was postponed. The infusion protocols were decided for each malignancy by the oncologists. The malignancies and HAI chemotherapy regimen varied across patients. Consequently, no survival analysis was performed in this study.

The 32 patients with secondary liver tumors (metastases from colorectal cancer (*n* = 30), ovarian cancer (*n* = 1) or squamous cell carcinoma of the anal canal (*n* = 1)) had raltitrexed/oxaliplatin combination (TOMOX) as first-line HAI chemotherapy, and the 11 patients with primary liver tumors (cholangiocarcinoma (*n* = 11)) had gemcitabine/oxaliplatin combination (GEMOX) as first-line HAI chemotherapy. A cycle was given every 3 weeks for TOMOX and every 2 weeks for GEMOX. All patients received at least one cycle of TOMOX or GEMOX (median, 4; range, 1–16). Patients were re-evaluated after every third cycle when possible and chemotherapy was continued up to tolerance or disease progression.

### 2.5. Data Collection and Definitions

Catheter dysfunction: catheter migration, occlusion and catheter implantation failure were recorded. We compared the frequency of catheter migration across the three catheter placement techniques. Catheter occlusion was treated by the perfusion of Alteplase 20 mg (Actilyse, Boeringer Ingelheim France, Paris, France). If catheter migration occurred, a new procedure to correct the position of the catheter was attempted. If extrahepatic perfusion was depicted on the control CT scan or the patient experienced abdominal pain related to chemotherapy infusion, a new arteriography was performed to look for an extrahepatic arterial branch. If such a branch was visualized, then its embolization was attempted using coils or NBCA or both.

Technical success: primary technical success was defined as implantation of the port-catheter. Secondary technical success was defined as administration of the chemotherapy, either after the procedure or after revision for abdominal pain related to HAI or for extrahepatic perfusion visualized by CT. 

Complications: complications reported during the follow-up period were classified retrospectively based on clinical relevance. Minor and major complications were categorized according to the Society of Interventional Radiology (SIR) classification [11]. Minor complications required no treatment or resolved within 24 h with symptomatic drugs. Major complications required treatment for more than 24 h or were more severe or irreversible [11]. We distinguished complications due to the arterial puncture from technical or mechanical complications due to the catheter implanted in the hepatic artery. The timing of these complications was categorized as either early, (within 1 month of the procedure), or late (more than 1 month after the procedure). The complication was considered resolved if its treatment allowed the use of the port catheter for HAI. Chemotherapy-related adverse events were not consistently reported. In the event of complications such as unmanageable catheter migration, unmanageable catheter occlusion, or infection unresponsive to antibiotics, the port catheter was removed under local anesthesia. 

### 2.6. Statistical Analysis

Values are described as mean ± SD (range) for normally distributed quantitative variables and as N (%) for qualitative variables. Comparisons of outcomes across the three patient groups managed with the three different port catheter placement methods were performed using Fisher’s exact test. Statistical significance was established for *p* values less than 0.05.

## 3. Results

### 3.1. Patient Characteristics

From October 2010 to August 2018, 43 patients underwent catheter-port system placement for HAI (30 men and 13 women; mean age, 58 years; age range, 40–80 years). Among them, 32 (74.4%) had secondary liver tumors and 11 (25.6%) had primary liver tumors. Of the 32 patients with secondary tumors, 30 (30/43, 69.8%) had metastases of a colorectal cancer, 1 had metastases of ovarian cancer, and 1 had metastases from squamous cell carcinoma of the anal canal. All 11 patients with primary tumors had cholangiocarcinomas. In all patients, the liver lesions were deemed unresectable and considered to be the survival-limiting factor. Table 1 reports the main patient characteristics including the variants in hepatic arterial anatomy. A classic celiac trunk was present in 30 patients (69.8%).

### 3.2. Primary Technical Success

**Figure 6:**
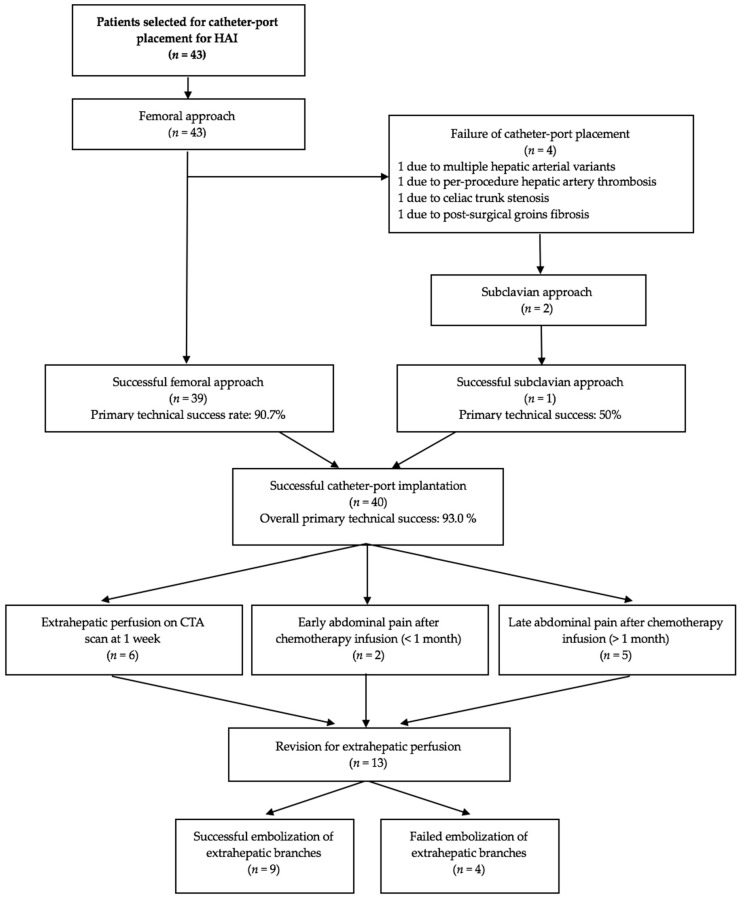
Flow chart of the study. HAI, hepatic arterial infusion; CTA, computed tomography angiography.

The femoral approach was successful in 39 (90.7%) patients. The 4 failures were due to fibrosis of both groins secondary to femoro-femoral bypass surgery (*n* = 1), celiac artery stenosis due to median arcuate ligament compression (*n* = 1), occlusion of the hepatic artery during the catheter placement procedure (major complication, *n* = 1), and multiple variants with a high risk of thrombosis (*n* = 1). This last patient had a right hepatic artery arising from the superior mesenteric artery, a left hepatic artery arising from the coronary artery of the stomach, and a middle hepatic artery arising directly from the aorta and giving rise to the gastroduodenal artery, which also supplied segment IV. The subclavian approach was attempted for two of these patients and was successful for 1. Thus, the overall primary technical success rate was 93.0%. For the 3 patients in whom a catheter could not be implanted, alternative techniques were used including systemic chemotherapy or another type of locoregional therapy such as transarterial radioembolization. Figure 6 is the flow chart.

### 3.3. Complications

Mean follow-up of the catheter-port systems was 140 days (range, 0–425 days). Table 2 reports the incidences of procedure-related complications, catheter dysfunction, and perfusion abnormalities. Complications occurred in 10 (25.0%) patients, including 6 with major and 4 with minor complications.

Major complications occurred in 6 (15.0%) patients. Among them, 2 patients developed infection a few months after catheter placement, with 1 of them having sepsis. The other patient required surgery for cellulitis and skin necrosis due to chemotherapy extravasation secondary to disconnection of the port from the catheter. Hepatic artery thrombosis occurred in 1 patient during catheter implantation, which therefore could not be achieved. Hematoma requiring percutaneous drainage occurred in 1 patient. Finally, 2 patients experienced gastroduodenal ulcers a few months after chemotherapy initiation. The diagnosis was suspected based on chronic abdominal pain and confirmed by endoscopy. In 1 of these patients, embolization of an ectopic artery was performed, allowing pain resolution and HAI resumption. In the other patient embolization of the extrahepatic branch failed, and HAI was stopped due to persistent abdominal pain despite medical management. No deaths were related to the procedure and no patients had femoral artery thrombosis.

Minor complications occurred in 4 (10.0%) patients, within 1 month after catheter placement. One patient had bleeding at the puncture site that stopped with manual compression. Small hematomas developed in 2 patients. Finally, non-occlusive dissection of a superior mesenteric artery occurred in 1 patient but did not require treatment.

Regarding catheter dysfunction, the port catheter became occluded in 2 patients. In 1 patient, the occlusion occurred 3 months after catheter placement and was successfully treated by the perfusion of Alteplase 20 mg (Actilyse^®^). The other patient had catheter occlusion after 18 months and required exchange of the occluded device by another port catheter. Extrahepatic perfusion was seen within 1 month of catheter implantation in 8 (18.6%) patients. The diagnosis was established by the 1-week follow-up CT scan in 6 patients and was discovered in the other 2 patients upon evaluation for abdominal pain. Embolization of the artery responsible for extrahepatic perfusion was successful in 6 patients. More than 1 month after catheter placement, extrahepatic perfusion was diagnosed in 5 patients due to abdominal pain. Embolization of the artery responsible for the extrahepatic perfusion was successful in 3 patients. The overall revision rate to treat extrahepatic perfusion was 13/40 (32.5%). The revision rate was significantly higher with the one-stage procedure than the two-stage procedure (36.0% vs. 26.7%, *p* = 0.042) (Table 3). Infusion hole migration occurred for 7 (17.5%) patients: 6 (37.5%) of the 16 patients with technique 1 and 1 (5.5%) of the 18 patients with technique 2. No catheter migration occurred with technique 3 (6 patients). However, the differences between technique 3 and the others was not statistically significant (*p* = 0.067). Table 3 reports the catheter revision and migration data.

### 3.4. Clinical and Secondary Technical Success

Three months after catheter implantation, 30 (69.8%) patients had received HAI. Embolization of the artery responsible for extrahepatic perfusion was possible in 9/13 (69.2%) patients. The catheter could not be implanted in 3 of the 43 patients, and 3 patients were lost to follow-up because they came from other centers. Two patients died. Intraarterial chemotherapy was postponed or interrupted because of major complications in 3 patients.

## 4. Discussion

This retrospective single-center study including 43 patients demonstrates a high primary technical success rate of 93.0% for percutaneous port-catheter placement. However, revision was necessary in 17.5% of patients due to catheter migration and in 32.5% due to extrahepatic perfusion. Revision for extrahepatic perfusion was successful in 9 (69.2%) of 13 patients. No catheter migration occurred with technique 3, but there were only 6 patients in this group. However, no significant differences were observed across the three catheter placement techniques. Finally, 69.8% of patients received HAI. Major complications occurred in 15%, with the majority being manageable. The two-stage procedure was associated with a significantly lower revision rate for extrahepatic perfusion.

This study confirms that, compared to surgical implantation, percutaneous implantation of port catheter systems is an easier and safer procedure that does not require general anesthesia [12,13]. Complication rates are lower than with surgically implanted systems [12]. We also show that radiologic placement can be carried out in patients with anatomic vascular variations, such as a hepatomesenteric trunk [6]. In contrast to the surgical method, catheter-port systems placed percutaneously cause less morbidity in the event of dysfunction, because the systems can be repositioned more easily [9]. Complicated surgical revisions or corrections requiring laparotomy can be avoided [9]. On the other hand, the 2 techniques may give similar outcomes out of dysfunction. Catheter-port systems are associated with various complications, such as occlusion and infection [14,15]. The relatively low complication rate with percutaneous implantation improves patient acceptance of the procedure [15]. Placement of the port catheter system on the anterior surface of the thigh below the groin seemed well accepted, even by very active patients. The femoral artery was chosen for implantation of the catheter-port system in our study because of its superficial location and easy access for puncture. The results indicate that percutaneous implantation of a port catheter system via the femoral artery is very safe, and our success rate was high, in keeping with earlier reports [1,5,16]. Studies have shown that subclavian access can generate a greater number of local complications due to the arterial puncture such as hematoma, bleeding, pseudoaneurysm, and arteriovenous fistula. In exceedingly rare cases, the subclavian approach can lead to pneumothorax or cerebrovascular accidents [17,18]. The subclavian approach should therefore be used only when the femoral approach is not feasible. 

Infection is another complication of permanently implanted catheter systems that often requires removal of the device. Infection and sepsis during chemotherapy can be caused by inadequate hygiene and can be treated successfully with antibiotics [16]. No prophylactic antibiotics were administered before the procedure in our study. Infection rates after radiologic implantation are lower than those after surgical implantation [9]. Therefore, interventional radiology suites seem to provide sufficient hygienic conditions to prevent infection when this type of intervention is performed. Thanks to the routine embolization of the right gastric artery and gastroduodenal artery [19,20] and to the use of CT to detect extrahepatic perfusion, we had only 2 cases of gastroduodenal ulcers [11]. No correlation was shown between the use of the side-hole catheter placement method (Technique 2) and a reduction in catheter migration [21,22]. These results do not confirm the findings shown in the study by Deschamps and al. [1], perhaps due to the smaller number of patients in our study. 

The two-stage procedure reduced the number of revisions to treat extrahepatic perfusion in our study. New extrahepatic arterial branches may become visible after occlusion of the gastroduodenal and right gastric arteries and can then be managed during the second stage. Another advantage of proceeding in two stages is a shorter time for each stage, which avoids radiologist fatigue and is more acceptable to the patients. Overall survival and progression-free survival were not evaluated in this study. Studies have already shown that HAI chemotherapy has a positive effect on overall survival and progression-free survival of patients who have unresectable metastases from colorectal cancer. In some cases, it is effective in downstaging the cancer, thereby allowing surgical resection [23].

We did not study the side effects of chemotherapy, as the literature is replete with data on this point [24,25]. In some cases, anatomical variations can be an obstacle to catheter implantation. As many as 25% of our patients experienced complications, although most of these resolved. Moreover, revision rates for extrahepatic perfusion (32.5%) and catheter migration (17.5%) were not negligible. Thus, percutaneous catheter implantation results in one or more repeat procedures in a substantial number of patients, generating constraints for the patients and radiologists. Yttrium-90 microspheres (SIR-Spheres; SIRTEX Medical, Boston, MA) are also well established as a well-tolerated treatment option for primary and secondary hepatic malignancies [4,26,27]. It may be of interest to compare HAI chemotherapy vs. yttrium-90 in terms of effectiveness, complication rates, and patient tolerance. All these points deserve to be explored in greater detail, particularly as the percutaneously implantable catheter-port system has more advantages than disadvantages.

Our study has several limitations. First, it has the limitations inherent in the retrospective single-center design. Second, the populations, malignancies, and chemotherapy regimens were heterogeneous, precluding an analysis of survival. Furthermore, some complications may have been related to the type of chemotherapy used. Third, technique 3 was used in only 6 patients. Finally, the number of patients in our study is considerably smaller than in other studies on the same topic.

## 5. Conclusions

In conclusion, our results demonstrate that percutaneous catheter placement performed under local anesthesia without a laparotomy is highly successful and safe. Our high technical success rate (93.0%), low complication rate, and acceptable time needed for the procedure (particularly with the two-stage method) suggest that this technique may deserve a central place in the treatment of unresectable liver metastases and primary liver cancer. Further studies in larger cohorts are warranted to better document the safety and to assess the long-term outcomes of this endovascular procedure.

## Figures and Tables

**Table 1:** Baseline characteristics of the 43 patients with liver cancer and procedural details.

Variable	Mean ± SD (Range) or N (%)
Age, years	58.3 ± 9.4 (40–80)
Sex	
Men	30 (69.8)
Women	13 (30.2)
Underlying malignancy	
Colorectal cancer	30 (69.8)
Cholangiocarcinoma	11 (25.6)
Ovarian cancer	1 (2.3)
SCC of the anal canal	1 (2.3)
Hepatic artery anatomy	
Classic celiac trunk	30 (69.8)
RHA	2 (4.7)
LHA	4 (9.3)
RHA + LHA	2 (4.7)
RHA + LHA + MHA	1 (2.3)
Other variants	4 (9.3)
Catheter-port implantation approach	
Technique 1 (free tip)	16 (40)
Technique 2 (fixed tip)	18 (45)
Technique 3 (back-up)	6 (15)

N, number; SCC, squamous cell carcinoma; RHA, right hepatic artery; LHA, left hepatic artery; MHA, middle hepatic artery. The data are mean ± SD (range) or N (%).

**Table 2:** Complications in the 43 patients with attempted implantation of a catheter-port system.

Type of Complication	Overall	Early (<1 Month)	Late (>1 Month)
	N (%)	N Overall/N Salvaged	N Overall/N Salvaged
Minor complications	4 (9.3)	4/4	0/NA
Small groin hematoma	3 (7.0)	3/3	0/NA
Arterial dissection *	1 (2.3)	1/1	0/NA
Major complications	6 (14.0)	2/1	4/2
Arterial thrombosis **	1 (2.3)	1/0	0/NA
Port-site infection	2 (4.7)	0/NA	2/1
Gastrointestinal ulcer	2 (4.7)	0/NA	2/1
Major groin hematoma ***	1 (2.3)	1/1	0/NA
Pump & catheter malfunction	22 (51.2)	15/12	7/4
Occlusion	2 (4.7)	0/0	2/1
Migration	7 (16.3)	7/6	0/NA
Extrahepatic perfusion	13 (30.2)	8/6	5/3
Procedure-related mortality	0 (0)	0/NA	0/NA

* Non-occlusive dissection of the superior mesenteric artery; ** Asymptomatic thrombosis of the proper hepatic artery; *** Groin hematoma requiring surgery; NA, not applicable. Data are expressed as N (%).

**Table 3:** Catheter-port system revision and migration rates in the 40 implanted patients.

Variable	N (%)
Revision rate by procedure type	13/40 (32.5)
One-stage procedure	9/25 (36.0)
Two-stage procedure	4/15 (26.7)
Migration rate by technique	7/40 (17.5)
Technique 1	6/16 (37.5)
Technique 2	1/18 (5.6)
Technique 3	0/6 (0)

Data are expressed as N (%).

## Data Availability

The data presented in this study are available on request from the corresponding author. The data are not publicly available due to identity reasons.
